# Expression of connexins and pannexins in diseased human liver

**DOI:** 10.17179/excli2022-5163

**Published:** 2022-08-22

**Authors:** Kaat Leroy, Vânia Vilas-Boas, Eva Gijbels, Bart Vanderborght, Lindsey Devisscher, Bruno Cogliati, Bert Van Den Bossche, Isabelle Colle, Mathieu Vinken

**Affiliations:** 1 Department of Pharmaceutical and Pharmacological Sciences, Entity of In Vitro Toxicology and Dermato-Cosmetology, Vrije Universiteit Brussel, Laarbeeklaan 103, 1090 Brussels, Belgium; 2 Department of Basic and Applied Medical Sciences, Gut-Liver Immunopharmacology Unit, Universiteit Gent, Corneel Heymanslaan 10, 9000 Gent, Belgium; 3 Department of Pathology, School of Veterinary Medicine and Animal Science, University of São Paulo, Av. Prof. Dr. Orlando Marques de Paiva 87, Cidade Universitária, 05508-270, São Paulo, Brazil; 4 Department of Hepatobiliary and Pancreatic Surgery, Algemeen Stedelijk Ziekenhuis Campus Aalst, Merestraat 80, 9300 Aalst, Belgium; 5 Department of Hepatology and Gastroenterology, Algemeen Stedelijk Ziekenhuis Campus Aalst, Merestraat 80, 9300 Aalst, Belgium

**Keywords:** connexin, pannexin, human liver disease, biopsies

## Abstract

Connexin proteins can form hexameric hemichannels and gap junctions that mediate paracrine and direct intercellular communication, respectively. Gap junction activity is crucial for the maintenance of hepatic homeostasis, while connexin hemichannels become particularly active in liver disease, such as hepatitis, fibrosis, cholestasis or even hepatocellular carcinoma. Channels consisting of connexin-like proteins named pannexins have been directly linked to liver inflammation and cell death. The goal of the present study was to characterize the expression and subcellular localization of connexins and pannexins in liver of patients suffering from various chronic and neoplastic liver diseases. Specifically, real-time quantitative reverse transcription polymerase chain reaction, immunoblotting and immunohistochemistry analyses were performed on human liver biopsies. It was found that pannexin1 and pannexin2 gene expression are correlated to a certain degree, as is pannexin1 protein expression with connexin32 and connexin43 protein expression. Furthermore, this study is the first to detect pannexin3 in human patient liver biopsies *via* both immunoblot and immunohistochemistry.

## List of abbreviations

ACTB Actin beta

ANOVA Analysis of variance

B2M Beta-2-microglobulin

CRLM Colorectal metastasis

Cx Connexin 

DAPI 4′,6-diamidino-2-phenylindole

F Female

G1 or G2 Glycosylated isoforms of Panx3

GAPDH Glyceraldehyde-3-phosphate dehydrogenase

GJA1 (Cx43) Gap junction protein alpha 1

GJB1 (Cx32) Gap junction beta 1 

GJB2 (Cx26) Gap junction beta 2

GJIC Gap junction intercellular communication

HCC Hepatocellular carcinoma

HMBS Hydroxymethylbilane synthase

M Male

NG Non-glycosylated 

NP Non-phosphorylated

p Probability 

P1 or P2 Phosphorylated isoforms of Cx43

Panx Pannexin 

PBS Phosphate buffered saline

PBS/T 1 % Triton X-100 dissolved in phosphate buffered saline

RT-qPCR Real-time quantitative re-verse transcription polymer-ase chain reaction

SD Standard deviation

UBC Ubiquitin C

## Introduction

The liver is responsible for the production of bile, synthesis of plasma proteins and xenobiotic detoxification amongst many other vital functions (Kalra et al., 2022[25]). One of the protein families involved in several of these critical functions is the group of connexins (Cx) (Maes et al., 2014[36]). These proteins consist of 4 transmembrane regions, 2 extracellular loops, and an intracellular N-terminus and C-terminus (Aasen et al., 2018[1]; Goodenough et al., 1996[21]; Nielsen et al., 2012[45]). Six Cx proteins can form a hexameric channel, called a Cx hemichannel, which allows the passage of small hydrophilic substances, such as ions and adenosine triphosphate, between the intracellular compartment and the extracellular space (Maes et al., 2014[36]; Nielsen et al., 2012[45]). When 2 hemichannels from neighboring cells dock, the resulting channel is called a gap junction and the flux of messenger molecules through these channels is denoted as gap junction intercellular communication (GJIC) (Nielsen et al., 2012[45]). Human liver harbors 3 main Cx isoforms, namely Cx26, Cx32 and Cx43 (Neveu et al., 1995[44]). Cx32 is the primary Cx variant being expressed by hepatocytes, while Cx43 is produced by non-parenchymal cells, such as stellate cells and Kupffer cells (Eugenin et al., 2007[14]; Fischer et al., 2005[17]; Neveu et al., 1995[44]). Cx26 is also expressed by hepatocytes and is mainly found in the periportal zone (Neveu et al., 1995[44]). Functional GJIC is necessary for hepatic homeostasis, yet many studies have shown the involvement of Cx proteins in liver disease, such as cholestasis (Fallon et al., 1995[15]; Gonzalez et al., 2002[20]), liver inflammation (Correa et al., 2004[11]; Nakashima et al., 2004[42]), fibrosis (Cogliati et al., 2016[8]; Nakata et al., 1996[43]) and even hepatocellular carcinoma (HCC) (Krutovskikh et al., 1994[30]; Ogawa et al., 2012[46]). Generally, Cx32 and Cx26 protein levels become downregulated, while Cx43 is upregulated upon liver pathology (Hernandez-Guerra et al., 2019[22]). Pannexin (Panx) proteins are also involved in liver physiopathology (Ganz et al., 2011[19]; Willebrords et al., 2018[60]). Panx proteins have been discovered about 2 decades ago and topologically resemble Cx proteins (Michalski et al., 2020[40]; Panchin et al., 2000[47]). However, they do not form gap junctions. Rather, they only form heptameric channels connecting the intracellular and extracellular microenvironments, reminiscent of Cx hemichannels (Michalski et al., 2020[40]; Qu et al., 2020[52]). Three different Panx isoforms have been described so far in humans, namely Panx1-3 (Cooreman et al., 2019[10]). Panx1-3 have been reported in liver (Bruzzone et al., 2003[6]; Le Vasseur et al., 2014[31]; Li et al., 2008[32]; Penuela et al., 2007[48]; Willebrords et al., 2018[60]). Panx1 is expressed by both parenchymal and non-parenchymal cells (Willebrords et al., 2018[60]). Panx3 protein might have been detected in low amounts in mouse liver (Penuela et al., 2007[48]), while Panx2 protein expression has only been detected in rat hepatocytes and cultured human HCC cells, so far (Bruzzone et al., 2003[6]; Li et al., 2008[32]; Xie et al., 2015[63]). In order to gain further insight into the involvement of Cx-based and Panx-based (hemi)channels in liver disease, identification of their expression, localization and potential correlation with any pathology is warranted. The aim of the current study was therefore to characterize Cx and Panx expression in liver samples of clinical patients suffering from various diseases both at the transcriptional and the translational level and to correlate those findings with disease status.

## Materials and Methods

### Sample collection

From 2014 until 2019, human liver tissue samples from 71 patients were collected at the Algemeen Stedelijk Ziekenhuis in Aalst-Belgium (Table 1(Tab. 1)). Three liver samples were surgically removed *per* patient, namely for RNA extraction, protein extraction and immunohistochemistry analysis, respectively. Samples from patients with neoplastic diseases or cysts were derived from surrounding (non-tumoral) liver tissue. Clinical data were provided for each patient. Histopathological examination was performed for disease diagnosis and staging. Samples for protein extraction were snap-frozen in liquid nitrogen and stored at -80 °C. Samples for total RNA extraction were submerged in RNALater (Thermo Fisher Scientific, USA), snap-frozen in liquid nitrogen and stored at -80 °C. Samples for *in situ* immunostaining were fixed in 10 % formaldehyde or methacarn solution and paraffin-embedded to be stored at room temperature (15-25 °C). Samples were collected without age-related or gender-related restrictions and represent a variety of liver diseases, such as cysts, chronic hepatitis, HCC and liver metastasis of colorectal adenocarcinoma (CRLM). This study was approved by the “Commissie Medische Ethiek” of the Universitair Ziekenhuis Brussel, the Vrije Universiteit Brussel and the ethics committee of the Algemeen Stedelijk Ziekenhuis Aalst (approval number B.U.N. 143201421250; registration number Aalst 052). Written informed consent was obtained from all the donors.

### Real-time quantitative reverse transcription polymerase chain reaction analysis

Extraction of total RNA, including the determination of its yield, was performed as described previously (Maes et al., 2016[39]). As such, 1 µg of total RNA was converted to cDNA with an iScript™ cDNA Synthesis Kit (Bio-Rad, USA) on a MiniAmp Plus Thermal Cycler (Thermo Fisher Scientific, USA). Resulting cDNA was purified using a GenElute™ PCR Clean-Up Kit (Sigma, USA). Real-time reverse transcription polymerase chain reaction analysis (RT-qPCR) was performed as described elsewhere (Maes et al., 2016[39]). An overview of the target and housekeeping genes can be found in Table 2(Tab. 2). All samples were tested in duplicate. Efficiency was calculated based on a 1 in 5 serial dilution of pooled cDNA. A non-template control was included as negative control. Results were analyzed according to the Pfaffl method, which accounts for differences in primer efficiencies (Pfaffl, 2001[50]). Data were normalized to a pooled control sample that was loaded onto every RT-qPCR plate to account for plate-to-plate variation.

### Immunoblot analysis

Immunoblot analysis was performed as described previously (Willebrords et al., 2016[61]) with some modifications in the separation of the proteins. A total of 10 µl per mg of liver tissue of radio-immunoprecipitation assay buffer (Thermo Fisher Scientific, USA) supplemented with 1 % ethylenediaminetetraacetic acid (Thermo Fisher Scientific, USA) and 1 % protease/phosphatase inhibitor cocktail (Thermo Fisher Scientific, USA) was added to 20-50 mg of liver tissue*. *The lysate was homogenized by an electric homogenizer (ULTRA-TURRAX T25, IKA, Germany) and incubated on ice for 10 minutes. Supernatants were collected by centrifugation at 14000 x *g *for 15 minutes at 4 °C. The Pierce^TM^ BCA protein assay kit (Thermo Fisher Scientific, USA) was used to determine protein concentrations. Next, 25 µg of each sample were pooled to function as a mixed control sample on every gel. For immunoblotting, 50 µg of each sample and the pooled control sample, identified as P in the blots, were separated on a 12 % Mini-PROTEAN TGX Stain-Free™ precast gel (Bio-Rad, USA). Proteins were transferred onto nitrocellulose membranes (Bio-Rad, USA) with the Trans-Blot Turbo™ Transfer System (Bio-Rad, USA) after which total protein loading (Supplementary Figure 1) was visualized on a ChemiDoc^TM^ MP imaging system (Bio-Rad, USA). Subsequently, membranes were blocked for 1 hour at room temperature (15-25 °C) in Tris-buffered saline solution (20 mM Tris and 135 mM sodium chloride) with 5 % skimmed milk (Régilait, France) and 0.1 % Tween-20 (Sigma, USA). Membranes were incubated overnight at 4 °C with a primary antibody targeted against Cx26, Cx32, Cx43, Panx1, Panx2 or Panx3 diluted in blocking buffer (Table 3(Tab. 3)). Membranes were washed 3 times for 10 minutes and incubated with a secondary antibody diluted 1:1000 for Cx43 and 1:500 for all other proteins (P0448 Dako, Denmark) in blocking buffer for 1 hour at room temperature (15-25 °C). Membranes were washed and visualized with the Pierce™ ECL Western Blotting Substrate kit (Thermo Fisher Scientific, USA) on a ChemiDoc^TM^ MP imaging system (Bio-Rad, USA). Signals were analyzed with Image Lab 6.0.1 software (Bio-Rad, USA). Data were normalized to the total protein loading instead of a housekeeping protein (Aldridge et al., 2008[2]), and expressed as fold change relative to the corresponding signals in the pooled control sample. This pooled sample was added to every gel to account for the gel-to-gel variation.

### Immunohistochemistry analysis

Liver tissue samples were fixed in 10 % formaldehyde or methacarn (Thermo Fisher Scientific, USA) and embedded in paraffin (Thermo Fisher Scientific, USA). As such, 5 µm thick liver slices were first deparaffinized in xylene (Thermo Fisher Scientific, USA) and subsequently rehydrated in 100 % ethanol (Thermo Fisher Scientific, USA), 90 % ethanol and 70 % ethanol. Next, slices were rinsed with running tap water. Antigen retrieval was performed by heating the slices in the microwave for 10 minutes in citrate buffer (pH 6.0) (Thermo Fisher Scientific, USA). Slices were washed extensively with PBS and permeabilized with 1 % Triton X-100 (Sigma, USA) dissolved in PBS (PBS/T). Subsequently, slices were thoroughly washed in PBS again. Samples were blocked at room temperature (15-25 °C) with 1 % bovine serum albumin and 5 % donkey serum (blocking buffer) (Sigma, USA) for 45 minutes and incubated overnight at 4 °C with primary antibody diluted in blocking buffer (Table 3(Tab. 3)). Slices were then washed in PBS/T and incubated with Alexa Fluor 594 - AffiniPure Donkey Anti-Rabbit IgG diluted 1:200 (711-585-152, Jackson ImmunoResearch Laboratories, USA) in blocking buffer for 90 minutes at room temperature (15-25 °C). Slices were washed with double distilled water. Finally, nuclei were stained during the mounting of the coverslips with VECTASHIELD Antifade Mounting Medium containing 4′,6-diamidino-2-phenylindole (DAPI) (Vector Laboratories, USA). Detection was performed at 20× magnification on a Nikon Eclipse T*i*-S microscope (Japan).

### Histopathological examination 

Paraffin-embedded samples were sectioned into 5 μm thick sections with a Leica RM2145 rotary microtome (Leica Biosystems, Belgium). Liver sections were stained with Sirius Red (Sigma, USA) and the degree of fibrosis was assessed at 100× magnification on an Olympus BX41 microscope (Olympus, Belgium). Scoring was performed blinded by 2 independent researchers according to the scoring parameters used in the Universitair Ziekenhuis Gent (Supplementary Figure 2). “Pericellular fibrosis” indicated fibrosis in the parenchyma between the portal triads without the clear formation of fibrotic septa. Samples with clear fibrotic strands (fibrotic septa) were classified with “septal fibrosis”. Subclasses of pericellular and septal fibrosis were made based on the extent of the fibrosis and were named “minimal pericellular fibrosis”, “clear pericellular fibrosis” and “beginning septal fibrosis”, respectively. The final class contained all cirrhotic samples.

### Statistical analysis 

The number of biological (N) and technical replicates (n) are mentioned in the figure legends. Data were normalized to a pooled sample during RT-qPCR and immunoblot analyses and presented as mean + standard deviation (SD). Statistical analysis was performed with GraphPad Prism 9 software. Normality was tested with the Shapiro-Wilk test. Normally distributed data was analyzed with a parametric T-test or a 1-way analysis of variance (ANOVA) to compare 2 or more groups, respectively. In case of non-normality, the non-parametric Mann-Whitney test or the Kruskal-Wallis test was used to compare 2 or more groups, respectively. Correlation was assessed by means of the non-parametric Spearman's rank correlation coefficient. Significance levels are indicated as *p ≤ 0.05, **p ≤ 0.01, ***p ≤ 0.001, and ****p ≤ 0.0001.

## Results

### Characterization of connexin and pannexin gene expression in human liver samples based on RT-qPCR analysis

In humans, hepatocytes mainly express Cx32 along with small amounts of Cx26 (Neveu et al., 1995[44]; Zhang and Nicholson, 1989[65]). Hepatocytes also produce Panx1 and Panx2 and might even express Panx3 (Bruzzone et al., 2003[6]; Le Vasseur et al., 2014[31]; Li et al., 2008[32]; Penuela et al., 2007[48]; Xiao et al., 2012;[62] Xie et al., 2015[63]). However, Panx2 expression has only been observed in rat liver (Le Vasseur et al., 2014[31]; Li et al., 2008[32]) and cultured human HCC cells (Xie et al., 2015[63]). Cx43 was previously detected in non-parenchymal cells, such as stellate cells and Kupffer cells, but not in hepatocytes (Eugenin et al., 2007[14]; Fischer et al., 2005[17]; Hernandez-Guerra et al., 2019[22]). In pathological conditions, Cx expression patterns drastically change in the liver, including an increase in Cx43 abundance, while Cx26, but in particular Cx32, is decreased (Hernandez-Guerra et al., 2019[22]). In the present study, mRNA expression levels of Cx26, Cx32, Cx43, Panx1, Panx2 and Panx3 were investigated in human liver samples. With the exception of Panx3, mRNA of all Cx and Panx isoforms investigated was detected in most human liver samples (individual data not shown). Changes in gene expression were analyzed based on various categories, such as fibrosis score, type of cancer and sex. Seven fibrosis groups were distinguished based on the histological fibrosis grade, ranging from no fibrosis to cirrhosis (Supplementary Figure 2). 

**Figure 1:**
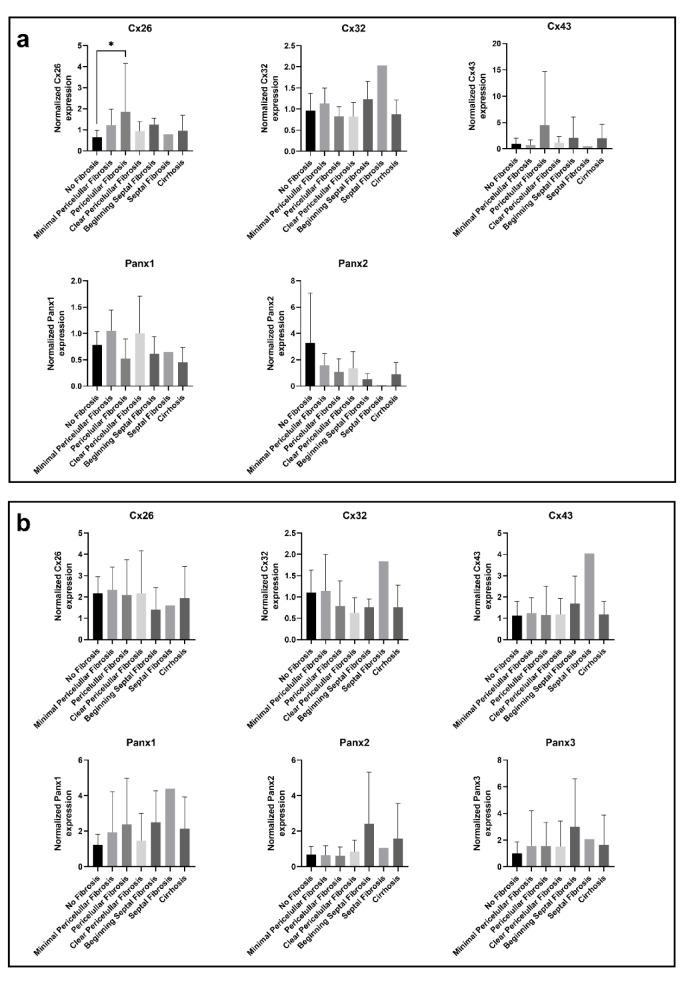
Cx and Panx gene (a) and protein (b) expression in human liver disease ranked by degrees of fibrosis. RT-qPCR analysis (a) and immunoblot analysis (b) of Cx26, Cx32, Cx43, Panx1, Panx2 and Panx3 was performed. Relative gene expression compared to a pooled sample was calculated with the Pfaffl method (Pfaffl, 2001). Protein levels were normalized to the total protein loading and expressed as a ratio to a pooled sample. Samples with varying levels of fibrosis (RT-qPCR: N = 1-14, n = 2; immunoblot: N = 1-14; n = 1) were compared with an ordinary one-way ANOVA or Kruskal-Wallis test depending on the normality of the data distribution. Graphs display data as mean + SD with * *p* ≤ 0.05.

**Figure 2:**
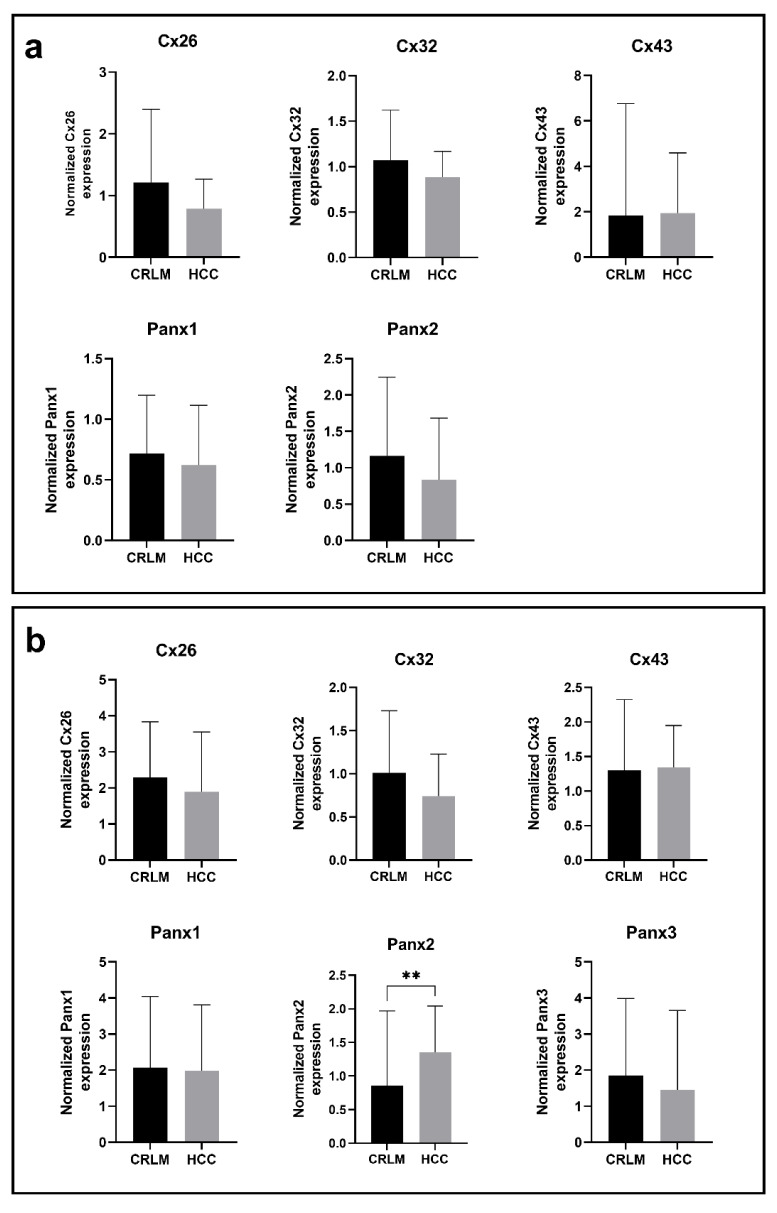
Cx and Panx gene (a) and protein (b) expression in CRLM and HCC samples. RT-qPCR analysis (a) and immunoblot analysis (b) of Cx26, Cx32, Cx43, Panx1, Panx2 and Panx3 was performed. Relative gene expression compared to a pooled sample was calculated with the Pfaffl method (Pfaffl, 2001). Protein levels were normalized to the total protein loading and expressed as a ratio to a pooled sample. CRLM samples (RT-qPCR: N = 42, n = 2; immunoblot: N = 43, n = 1) and HCC samples (RT-qPCR: N = 10, n = 2; immunoblot: N = 12, n = 1) were compared with a Mann-Whitney test or unpaired t-test depending on the normality of the data distribution. Graphs display data as mean + SD with ** *p* ≤ 0.01.

**Figure 3:**
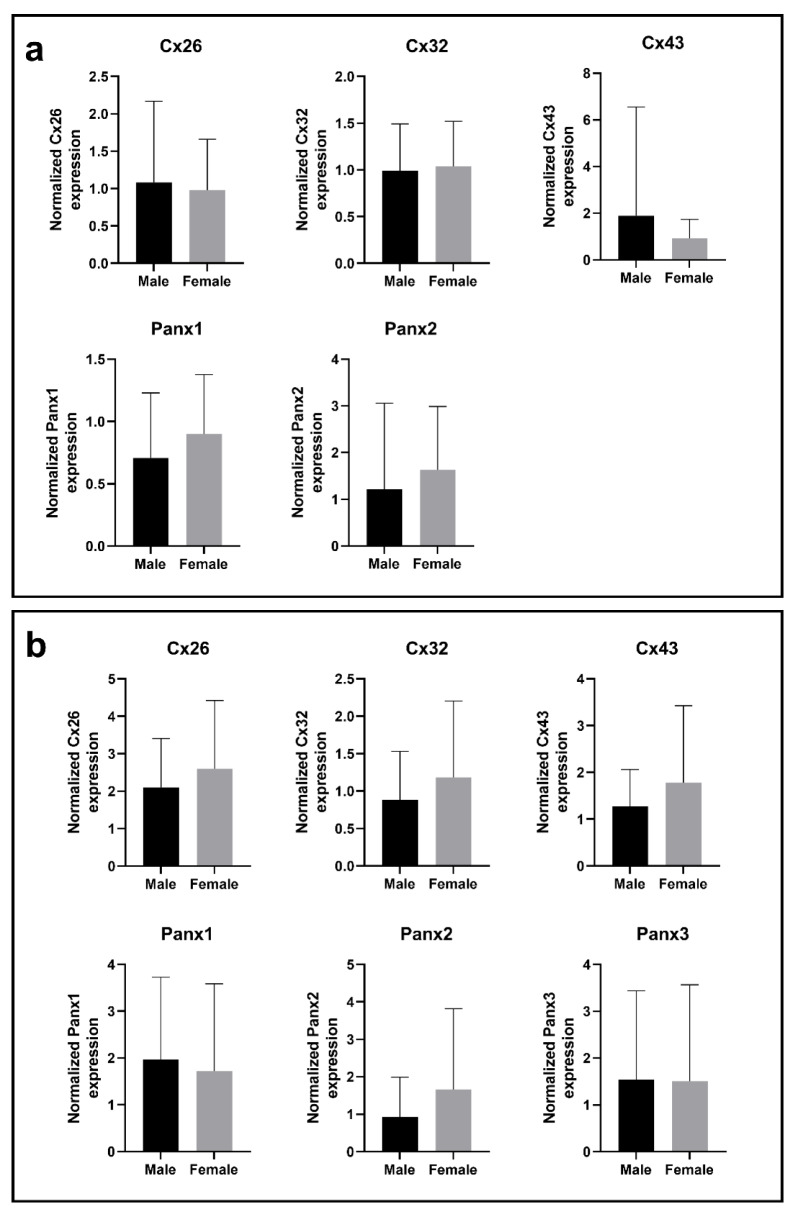
Cx and Panx gene (a) and protein (b) expression in male samples and female samples. RT-qPCR analysis (a) and immunoblot analysis (b) of Cx26, Cx32, Cx43, Panx1, Panx2 and Panx3 was performed. Relative gene expression compared to a pooled sample was calculated with the Pfaffl method (Pfaffl, 2001). Protein levels were normalized to the total protein loading and expressed as a ratio to a pooled sample. Male samples (RT-qPCR: N = 51, n = 2; immunoblot: N = 53; n = 1) and female samples (RT-qPCR: N = 16; n = 2; immunoblot: N = 16; n = 1) were compared with a Mann-Whitney test or unpaired t-test depending on the normality of the data distribution. Graphs display data as mean + SD.

During the analysis, Cx or Panx expression was routinely compared with the liver samples of patient without fibrosis. The mean expression levels were compared between CRLM and HCC samples to assess the Cx and Panx expression in 2 different types of cancer, while male and female samples were compared for the parameter “sex”. In this regard, Cx26 gene expression appears to be increased in samples with pericellular fibrosis compared to samples without fibrosis (Figure 1a(Fig. 1)). Cx32 gene expression also seems to be increased during septal fibrosis, but this fibrosis grade was not included into any ANOVA analysis because it only contained 1 sample. No other consistent changes in Cx or Panx expression could be observed within each of the categories (Figure 1-3 (a)(Fig. 1)(Fig. 2)(Fig. 3)).

### Characterization of connexin and pannexin protein expression in human liver samples based on immunoblot analysis

**Figure 4:**
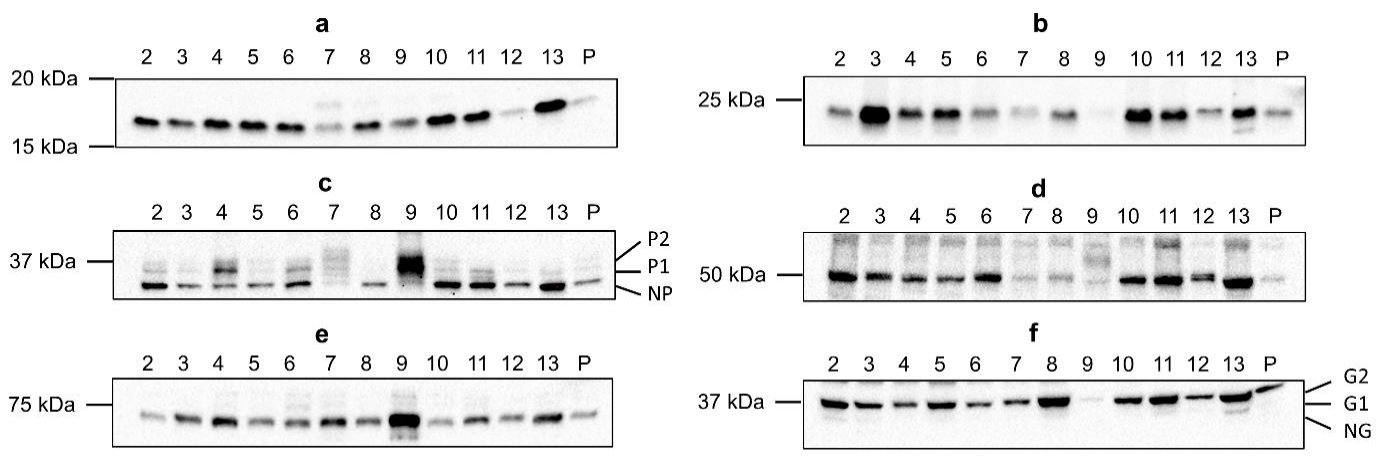
Cx26 (a), Cx32 (b), Cx43 (c), Panx1 (d), Panx2 (e) and Panx3 (f) protein expression in human liver disease. Total protein was extracted from the human liver biopsies (N = 70; n = 1) and used for immunoblotting analysis of all Cx and Panx protein targets. Immunoblots were visualized with a Pierce^TM^ ECL Western Blotting Substrate kit (Thermo Fisher Scientific, USA) on a ChemiDoc^TM ^MP imaging system (Bio-Rad, USA). A representative blot per protein target is shown. Sample numbers are indicated above the blot. (P, pooled sample; P1 and P2, phosphorylated isoforms; NP, non-phosphorylated isoform; G1 and G2, glycosylated isoforms; NG, non-glycosylated isoform)

Cx26 was detected around 17 kDa, while Cx32 was found right below 25 kDa (Figure 4(Fig. 4); Supplementary Figures 3-4). This lower-than-expected molecular weight could be related to the partial Cx proteolysis that takes place during protein extraction (Willebrords et al., 2016[61]). Cx43 displayed 3 bands at different molecular weights, representing the non-phosphorylated isoform (NP) and phosphorylated isoforms (P1 and P2) (Figure 4(Fig. 4); Supplementary Figure 5). In contrast to Cx26, Cx32 and Cx43 are both phosphoproteins (Zhang and Nicholson, 1989[65]). However, Cx32 phosphorylation cannot be detected *via* immunoblotting analysis (Willebrords et al., 2016[61]). Panx1 and Panx2 appeared around 50 kDa and 71 kDa, respectively (Figure 4(Fig. 4); Supplementary Figures 6-7). Panx3 was detected at a molecular weight of 38 kDa in most samples, but some samples also displayed one or two faint signals between 33 kDa and 37kDa (Figure 4(Fig. 4); Supplementary Figure 8). The signal at the lowest molecular weight represents the non-glycosylated (NG) isoform, while the second highest (G1) and highest signal (G2) reflect the high mannose and the complex glycoprotein isoform, respectively (Penuela et al., 2007[48], 2009[49]). Furthermore, as done for the mRNA analysis, changes in protein expression were analyzed based on fibrosis score, type of neoplastic disease (CRLM or HCC) and sex (Figure 1-3(b)(Fig. 1)(Fig. 2)(Fig. 3)). It was found that Panx2 is expressed to a lower extent in CRLM compared to HCC (Figure 2b(Fig. 2)). No other changes were noted.

### Characterization of the correlation between connexin and pannexin expression at the gene and protein level

**Figure 5:**
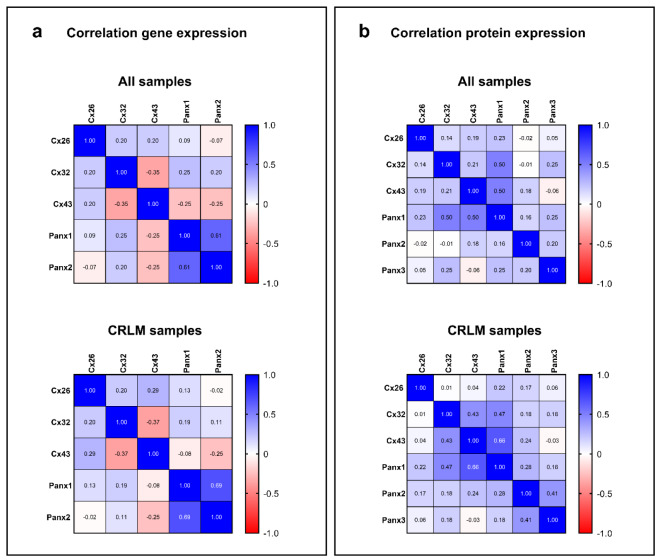
Correlation between Cx and Panx expression in human liver disease. The heat maps represent the Spearman's rank correlation coefficients between Cx and Panx gene (a) and protein (b) expression. Total RNA was extracted from the human liver biopsies and used for RT-qPCR analysis of Cx26, Cx32, Cx43, Panx1, Panx2 and Panx3. Relative fold gene expression compared to a pooled sample was calculated with the Pfaffl method (Pfaffl, 2001). Total protein was extracted from the human liver biopsies and used for immunoblotting analysis of Cx26, Cx32, Cx43, Panx1, Panx2 and Panx3. Protein levels were normalized to the total protein loading and expressed as a ratio to a pooled sample.

The Spearman's rank correlation coefficient was calculated to assess the correlation between Cx and Panx expression. A Spearman's rank coefficient measures the direction and the strength of the link between 2 variables (Al-Jabery et al., 2020[3]). This provided an indication of the association between the gene or protein expression of the targets of interest. Throughout all observations, Panx1 and Panx2 gene expression seemed to be moderately correlated with each other (Figure 5a(Fig. 5)) as evidenced by the significant Spearman correlation coefficient equaling 0.61 (*p* = 4,95E-08). This correlation even increased to 0.69 (*p *= 5.319E-007) when considering the expression of Panx1 and Panx2 in CRLM samples only. A correlation coefficient of 0.50 was noticed between Panx1 protein expression and both Cx43 (*p *= 9.922E-006) and Cx32 (*p *= 1.092E-005) protein expression (Figure 5b(Fig. 5)). When only considering CRLM patients, the correlation between Cx43 and Panx1 protein expression increased to 0.66 (*p* = 1.379E-006).

### Characterization of connexin and pannexin protein localization in human liver samples

**Figure 6:**
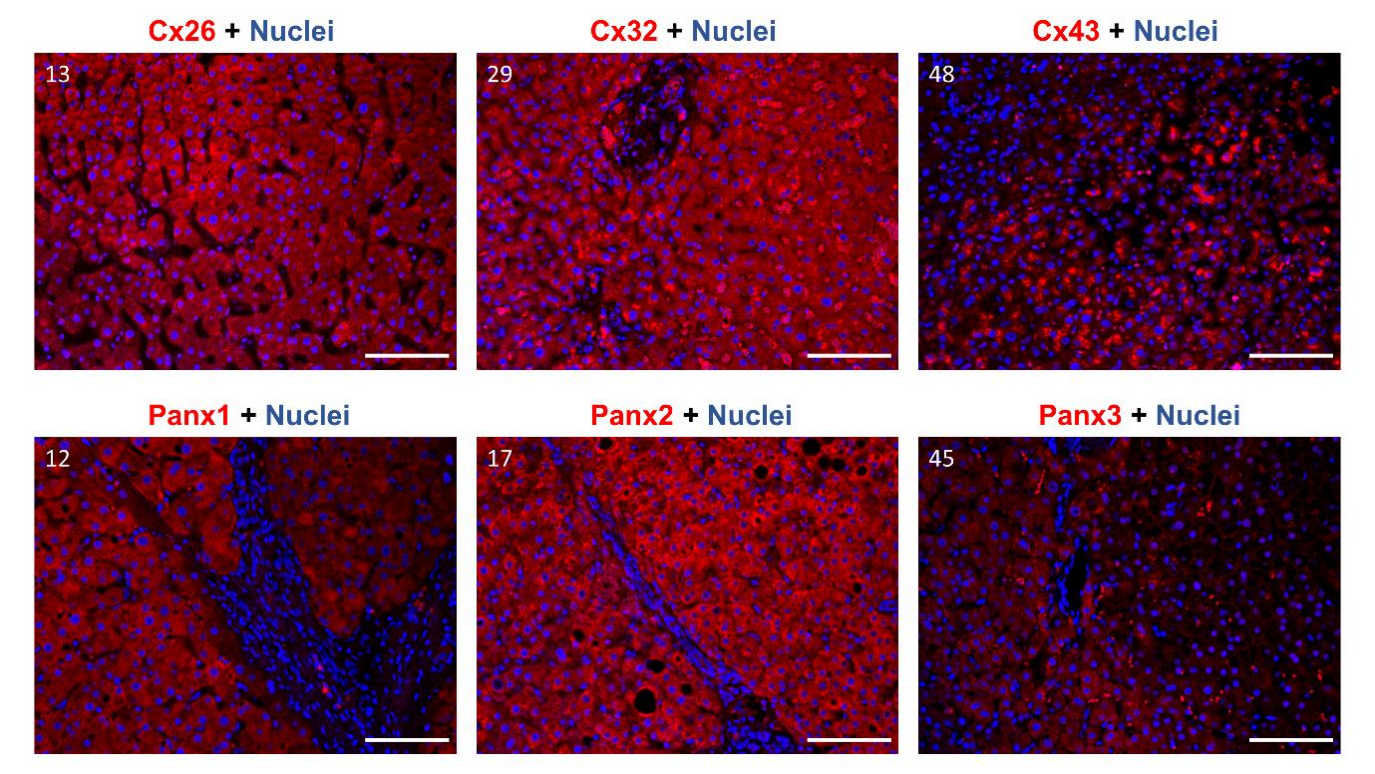
Cx26, Cx32, Cx43, Panx1, Panx2 and Panx3 protein localization in human liver samples. Samples were selected to undergo immunohistochemistry analysis based on the immunoblot results. Paraffin-embedded samples were sectioned into 5 μm thick sections. Cx26, Cx32, Cx43, Panx1, Panx2 and Panx3 are visualized in red, while the nuclei are counterstained in blue (DAPI). Detection was performed at 20× magnification. Scale bar = 100 µm. Sample numbers are indicated in the left upper corner of the images.

Gap junctions occupy approximately 3 % of the hepatocyte membrane (Maes et al., 2014[36]). Cx and Panx proteins typically reside in the cell plasma membrane (Epp et al., 2019[13]; Fort et al., 2011[18]; Nakashima et al., 2004[42]; Penuela et al., 2007[48]). Nevertheless, a substantial portion of a cell's Cx content can be detected in the cytoplasm, due to their rapid turn-over rate (Beardslee et al., 1998[4]; Chu and Doyle, 1985[7]; Fallon and Goodenough, 1981[16]; Maes et al., 2016[37]). Furthermore, a shift towards the cytoplasmic location is generally seen in pathological conditions for both Cx and Panx proteins (Beardslee et al., 1998[4]; Berthoud et al., 2004[4]; Fallon and Goodenough, 1981[16]; Hernandez-Guerra et al., 2019[22]; Kawasaki et al., 2007[26]; Maes et al., 2017[38]; Nakashima et al., 2004[42]). Panx2 is an exception to this and is mostly found in the cytoplasm, even in physiological conditions (Le Vasseur et al., 2014[31]). Immunohistochemistry analysis was performed to visualize subcellular location of Cx26, Cx32, Cx43, Panx1, Panx2 and Panx3 in human liver samples *in situ* (Figure 6(Fig. 6); Supplementary Figures 9-15). Based on the protein levels determined by immunoblot analysis, the 4 samples with the highest protein expression were selected per target protein. Images for each of these 4 samples can be found in the supplementary material (Supplementary Figures 9-15). One representative image per protein of interest is shown in Figure 6(Fig. 6). For Cx26, 3 of the highest expressing samples were derived from patients with CRLM (samples 13, 36 and 39). The fourth sample represents a biopsy from a patient with a liver metastasis of pancreatic adenocarcinoma (sample 61). The fibrosis score ranged from “minimal pericellular fibrosis” to “clear pericellular fibrosis” in these samples. Based on the fluorescent signal, Cx26 seems to be diffusely expressed in the cytoplasm of the hepatocytes (Supplementary Figure 9). Signals were usually evenly spread out in the samples of CRLM patients, but sample 61 containing the liver metastasis of pancreatic adenocarcinoma displayed an uneven Cx26 signal (Supplementary Figure 9). When performing a replicate staining on sample 13 (CRLM), a zonated pattern appeared across the liver sample (Supplementary Figure 10). The signal in the cytoplasm appeared to be intensified in one region compared to the other zones in the liver sample (Supplementary Figure 10), possibly indicating a difference in expression levels. Cx26 was also detected as a punctuated pattern in the zone with the intensified signal, hinting at a localization in the cell's plasma membrane. The 4 samples expressing the highest Cx32 levels originated from patients with CRLM displaying “no fibrosis” or “minimal to no fibrosis”. Although these samples share the same histopathology, the immunohistochemistry analysis revealed a very different expression pattern (Supplementary Figure 11). Sample 29 (CRLM) and sample 43 (CRLM) showed a diffuse signal across the cytoplasm of the hepatocytes together with some intensified punctuation around the vessel in sample 43. However, sample 52 (CRLM) and sample 58 (CRLM) lacked this diffuse cytoplasmic signal. This difference between the samples could not be attributed to a zonation pattern, since all samples displayed an even Cx32 signal across the slices and around the present vessels. In contrast to Cx26 and Cx32, the 4 biopsies containing the highest Cx43 expression were very diverse (Supplementary Figure 12). They represented 3 different diseases, namely CRLM, cholangiocarcinoma and hepatocellular adenoma. Additionally, they all displayed a different degree of fibrosis. The Cx43 signal appeared to be diffuse but evenly spread across the cytoplasm in the parenchymal cells of the liver. Similar diffuse cytoplasmic patterns were seen for Panx1, Panx2 and Panx3, which were also evenly detected across the hepatocytes in the liver biopsies (Figure 6(Fig. 6) and Supplementary Figures 13-15). However, the signal of Panx3 was less intense. The samples selected for the Panx immunohistochemistry analysis all had varying degrees of fibrosis.

## Discussion

It has been reported on many occasions that Cx and Panx (hemi)channels are involved in disease, especially by mediating communication related to inflammation (Cogliati et al., 2016[8]; Eugenin et al., 2007[14]; Fallon et al., 1995[15]; Ganz et al., 2011[19]; Hernandez-Guerra et al., 2019[22]; Krutovskikh et al., 1994[30]; Maes et al., 2017[38]; Nakashima et al., 2004[42]; Nakata et al., 1996[43]; Ogawa et al., 2012[46]; Willebrords et al., 2018[60]; Xiao et al., 2012[62]). However, less attention has yet been paid to the investigation of the fate of the building blocks of these (hemi)channels, namely Cx and Panx proteins, during disease, *in casu* liver pathology. In this respect, Cx32 and Cx26 protein levels are significantly downregulated *in vivo* in acute liver injury and cholestasis, while Cx43 levels increase (Cooreman et al., 2020[10]; Fallon et al., 1995[15]; Gonzalez et al., 2002[20]; Maes et al., 2016[37]; Sáez et al., 1997[53]). Downregulation of Cx32 is also seen in patients with hepatitis, cirrhosis and HCC (Nakashima et al., 2004[42]). Cx43 expression, on the other hand, can be upregulated or downregulated in HCC patients (Wilgenbus et al., 1992[59]; Yang et al., 2016[64]). Panx1 probably acts as a liver tumor promotor, since liver samples of patients with more advanced HCC express higher levels of Panx1 compared to liver samples of patients with less advanced HCC stages (Shi et al., 2019[55]). Additionally, Panx1 is involved in inflammation during acute and chronic liver disease, such as non-alcoholic steatohepatitis and hepatitis C, and inhibition of its channels leads to alleviation of acetaminophen-induced cytotoxicity *in vivo* (Ganz et al., 2011[19]; Kim et al., 2021[28]; Maes et al., 2017[38]; Willebrords et al., 2018[60]). Panx2 has been associated with breast cancer metastasis, clear renal cell carcinoma, prostate cancer or cholangiocarcinoma (Kim et al., 2019[27]; Liao et al., 2020[33]; Liu et al., 2019[34]; Qian et al., 2021[51]), while in liver it has been postulated as a tumor suppressor *in vitro* (Xie et al., 2015[63]). Finally, Panx3 has been linked to osteosarcoma and post-traumatic osteoarthritis (Moon et al., 2015[41]; Sun et al., 2020[56]), but its involvement in human liver disease has not yet been investigated. 

This study aimed to characterize the expression of all Panx family members in a vast array of liver-specific pathologies and liver metastases to establish any correlation with a number of parameters, including Cx expression, but also type of cancer, fibrosis score and sex. A moderate (Schober et al., 2018[54]), yet significant correlation between Panx1 and both Cx32 and Cx43 protein levels was found. Additionally, Cx26 gene expression was increased in samples with pericellular fibrosis compared to the samples without fibrosis. Interestingly, although Panx3 mRNA was not detected, Panx3 protein expression was demonstrated by 2 techniques, namely immunoblotting and immunohistochemistry analyses. In fact, immunoblot analysis could detect the non-glycosylated isoform, the high mannose isoform and the complex glycoprotein isoform of Panx3 (Penuela et al., 2009[49]). Similar, discrepancy between mRNA and protein levels has been reported earlier for Panx2 in the central nervous system and has been associated with the long half-life of Panx proteins (Diezmos et al., 2015[12]; Le Vasseur et al., 2014[31]; Penuela et al., 2007[48]), ranging from 17 to 100 hours (Chu and Doyle, 1985[7]). Additionally, it is often noted that transcript amounts cannot directly predict protein levels during stress responses (Liu et al., 2016[35]). To the best of our knowledge, Panx3 expression in liver has not yet been previously reported neither in healthy nor in pathological conditions in humans (Iwamoto et al., 2017[24]). This study is also the first to report the presence of Panx2 protein in human liver samples representing surrounding tissue of (non)neoplastic liver disease. Panx2 was previously detected as protein in rat liver or as mRNA in a human HCC cell line and a healthy human liver cell line (Bruzzone et al., 2003[6]; Li et al., 2008[32]; Xie et al., 2015[63]). Panx2 protein expression was differentially expressed in CRLM and HCC. Moreover, Panx2 and Panx1 gene expression levels were found to positively correlate (Schober et al., 2018[54]), which raises questions about the role previously assigned to Panx1 and Panx2. While Panx1 was found to act as liver tumor promotor, Panx2 rather performs tumor-suppressive actions in human HCC (Shi et al., 2019[55]; Xie et al., 2015[63]). Although Panx2 has been specifically detected in the lateral plasma membrane of hepatocytes (Li et al., 2008[32]), the present results suggest that Panx2 might be found in the intracellular compartment of hepatocytes in human biopsies of surrounding tissue from patients with different neoplastic diseases or various liver diseases. Panx2 has been observed in the cytoplasm of different tissues, such as cuboidal kidney tubule cells, neurons or germ cells, in contrast to the other Panx family members, which are typically located in the cell plasma membrane (Le Vasseur et al., 2014[31]). All other proteins subjected to immunohistochemistry analysis in this study also seemed to be located in the cytoplasm of the hepatocytes from the selected samples mainly surrounding tissue of CRLM. Although not the main area of expression (Epp et al., 2019[13]; Fort et al., 2011[18]; Nakashima et al., 2004[42]; Penuela et al., 2007[48]), Cx proteins can reside in the cytoplasm due to their short half-lives of merely 1 to 5 hours (Beardslee et al., 1998[4]; Berthoud et al., 2004[5]; Fallon and Goodenough, 1981[16]; Traub et al., 1987[58]). Furthermore, aberrant subcellular localization can be caused by liver disease. Multiple studies have reported a shift to the cytoplasm in disease conditions for both Cx and Panx proteins (Kawasaki et al., 2007[26]; Maes et al., 2017[38]; Nakashima et al., 2004[42]). It is suspected that Cx26 was partly located at the hepatocytes cell membrane in 1 zone of sample 13. Additionally, the expression of Cx26 seemed to be enhanced in this area compared to other areas of the same sample. Various studies have reported zonation for Cx26, where it is primarily found in periportal hepatocytes due to the presence of glucagon, which stabilizes Cx26 mRNA (Iwai et al., 2000[23]; Kojima et al., 1995[29]; Traub et al., 1989[57]). 

In summary, the presented data provide an overview of the Cx and Panx expression in human liver samples representing various pathologies. Based on this study, it seems that Panx1 and Panx2 gene expression can be correlated to a certain degree, as well as the protein expression of Panx1 and Cx32 and Cx43. Finally, Panx3 protein was detected for the very first time in human liver biopsies, opening new opportunities for future research on the role of Panx3 in the homeostatic or pathological processes of the liver.

## Declaration

### Conflicts of interest

The authors report no conflicts of interest.

### Acknowledgments

The authors wish to thank Miss Dinja De Win, Ms. Tâmara Prandini and Miss Yasmin Dahdouh-Guebas for their excellent technical assistance. The authors are particularly grateful to Mr. Paul Claes for transportation and storage of all human liver samples. This work was financially supported by the Research Foundation Flanders-Belgium (FWO Vlaanderen), the Marie Skłodowska-Curie Actions (grant 833095), the Fundação de Amparo à Pesquisa do Estado de São Paulo (13/50420-6; 18/10953-9), the Methusalem program of the Flemish government-Belgium and the University Hospital of the Vrije Universiteit Brussel-Belgium (Willy Gepts Fonds UZ-Brussel). 

## Supplementary Material

Supplementary information

## Figures and Tables

**Table 1:**
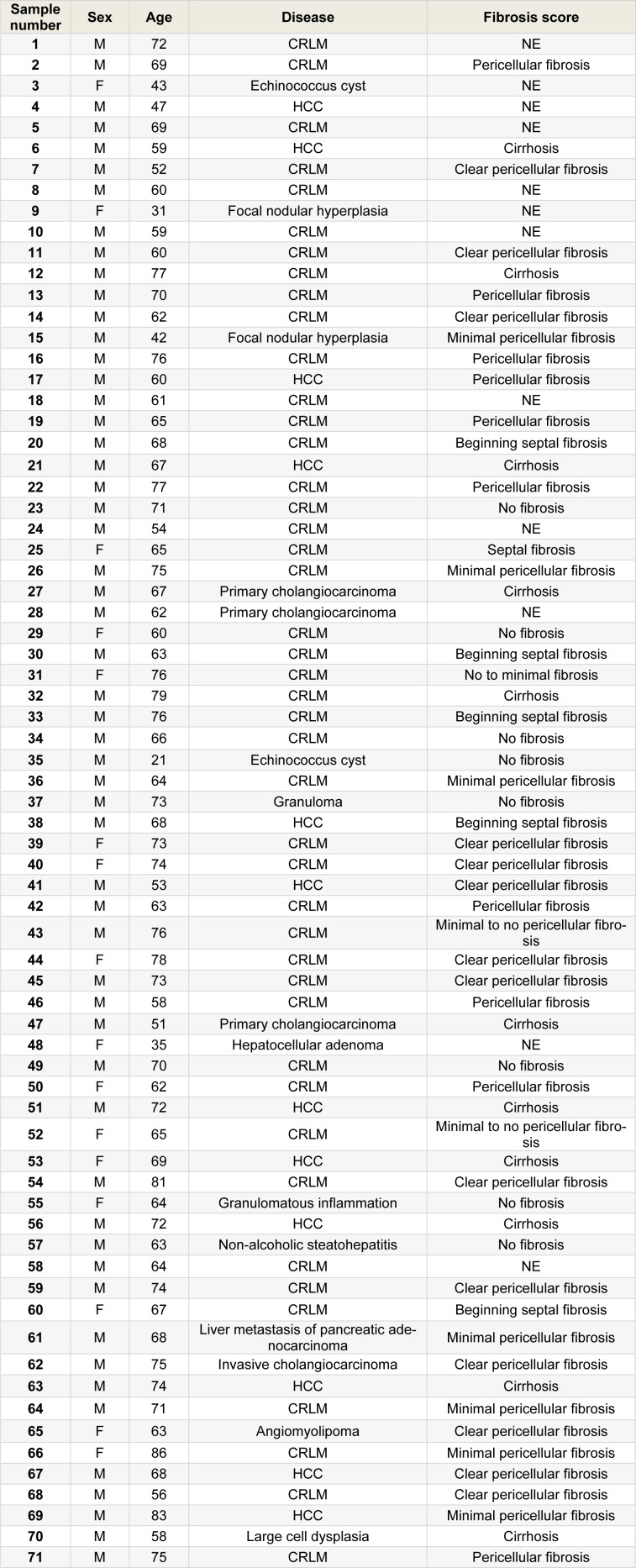
Overview of the human liver samples analyzed in this study. An overview of the sex, age, disease and fibrosis score of the donors is provided (M, male; F, female; CRLM, colorectal metastasis; HCC, hepatocellular carcinoma; NE, not evaluated).

**Table 2:**
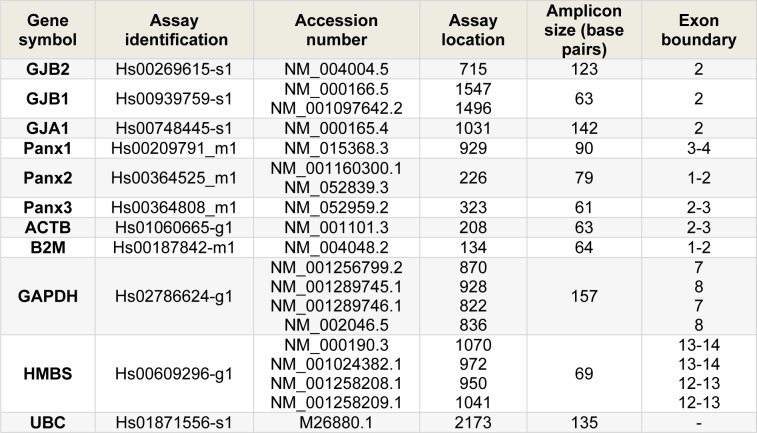
Primers and probes used in the RT-qPCR analysis. Assay identification, accession number, assay location, amplicon size and exon boundaries are listed (GJB2, Cx26; GJB1, Cx32; GJA1, Cx43; ACTB, actin beta; B2M, beta-2-microglobulin; GAPDH, glyceraldehyde-3-phosphate dehydrogenase; HMBS, hydroxymethylbilane synthase; UBC, ubiquitin C).

**Table 3:**
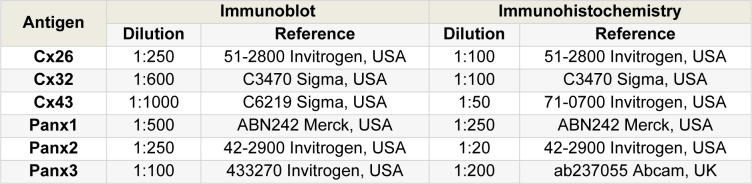
Primary antibodies used in immunoblotting and immunohistochemistry analysis. Dilution and reference of each antibody are presented.
