# Supplementary material for: Expression of connexins and pannexins in diseased human liver
Source: EXCLI J. 2022 Aug 22;21:1111–29. doi: 10.17179/excli2022-5163 (PMC9650699; doi:10.17179/excli2022-5163)
Supplement: Supplementary information [file EXCLI-21-1111-s-001.pdf]

EXCLI Journal 2022;21:1111-1129 – ISSN 1611-2156
Received: June 23, 2022, accepted: August 17, 2022, published: August 22, 2022

Supplementary information to:

Original article:

EXPRESSION OF CONNEXINS AND PANNEXINS IN DISEASED HUMAN LIVER

Kaat Leroy[a], Vânia Vilas-Boas[a #], Eva Gijbels[a], Bart Vanderborght[b], Lindsey Devisscher[b], Bruno Cogliati[c], Bert Van Den Bossche[d], Isabelle Colle[e], Mathieu Vinken[a]

[a] Department of Pharmaceutical and Pharmacological Sciences, Entity of In Vitro Toxicology and Dermato-Cosmetology, Vrije Universiteit Brussel, Laarbeeklaan 103, 1090 Brussels, Belgium
[b] Department of Basic and Applied Medical Sciences, Gut-Liver Immunopharmacology Unit, Universiteit Gent, Corneel Heymanslaan 10, 9000 Gent, Belgium
[c] Department of Pathology, School of Veterinary Medicine and Animal Science, University of São Paulo, Av. Prof. Dr. Orlando Marques de Paiva 87, Cidade Universitária, 05508-270, São Paulo, Brazil
[d] Department of Hepatobiliary and Pancreatic Surgery, Algemeen Stedelijk Ziekenhuis Campus Aalst, Merestraat 80, 9300 Aalst, Belgium
[e] Department of Hepatology and Gastroenterology, Algemeen Stedelijk Ziekenhuis Campus Aalst, Merestraat 80, 9300 Aalst, Belgium

[#] Current affiliation: International Iberian Nanotechnology Laboratory, Braga, Portugal

* **Corresponding author:** Prof. Mathieu Vinken (Ph.D., Pharm.D., E.R.T.), Department of Pharmaceutical and Pharmacological Sciences, Entity of In Vitro Toxicology and Dermato-Cosmetology, Vrije Universiteit Brussel, Laarbeeklaan 103, 1090 Brussels, Belgium; Tel.: +3224774587; E-mail: mathieu.vinken@vub.be

https://dx.doi.org/10.17179/excli2022-5227

This is an Open Access article distributed under the terms of the Creative Commons Attribution License (http://creativecommons.org/licenses/by/4.0/).

EXCLI Journal 2022;21:1111-1129 – ISSN 1611-2156
Received: June 23, 2022, accepted: August 17, 2022, published: August 22, 2022

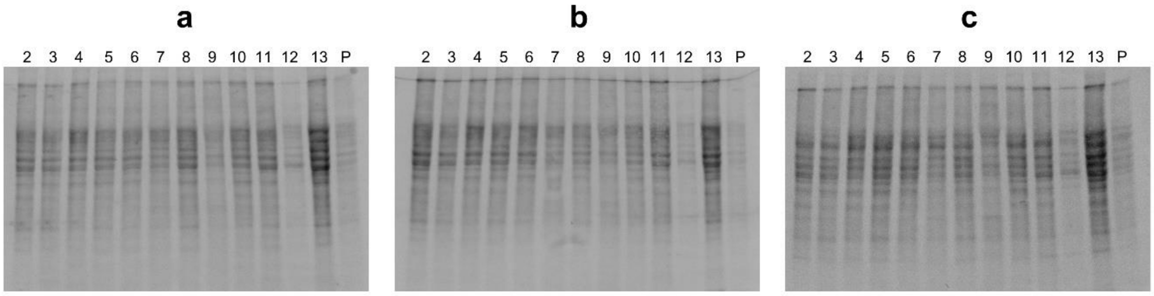

Supplementary Figure 1: Images of total protein loading for normalization during immunoblot analysis. Total protein was extracted from the human liver biopsies (N = 70; n = 1) and used for immunoblotting analysis. Immunoblots were visualized on a ChemiDoc^{TM} MP imaging system (Bio-Rad, USA) before treatment with antibodies. This image allows quantification of the total amount of proteins that is loaded per sample. Total protein loading was used for normalization purposes instead of a housekeeping protein. Representative images are shown. Sample numbers are indicated above the blot. (P, pooled control sample). To save sample material, target proteins were detected on the same immunoblot when the molecular weight allowed clear separation of the signal. Total protein loading of the first 13 samples is shown for Cx43 (**a**), Panx1 and Cx32 (**b**) and Panx2/3 and Cx32 (**c**).

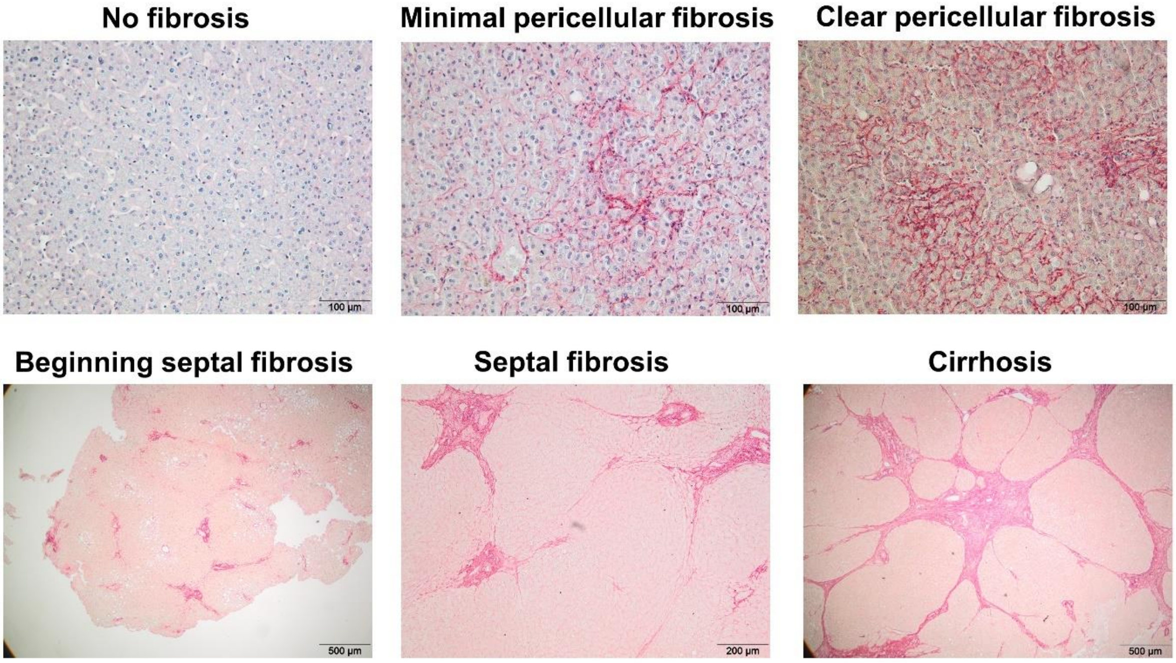

Supplementary Figure 2: Overview of fibrosis degrees. Human liver samples were divided into different categories based on the fibrosis grade. Four major classifications can be distinguished, namely "no fibrosis", "pericellular fibrosis", "septal fibrosis" and "cirrhosis". Based on the extent of the fibrosis, subclasses were made for "pericellular fibrosis" and "septal fibrosis". Representative images of the different fibrotic stages are shown in this figure at varying magnifications. Scale bars are indicated in the respective image.

EXCLI Journal 2022;21:1111-1129 – ISSN 1611-2156
Received: June 23, 2022, accepted: August 17, 2022, published: August 22, 2022

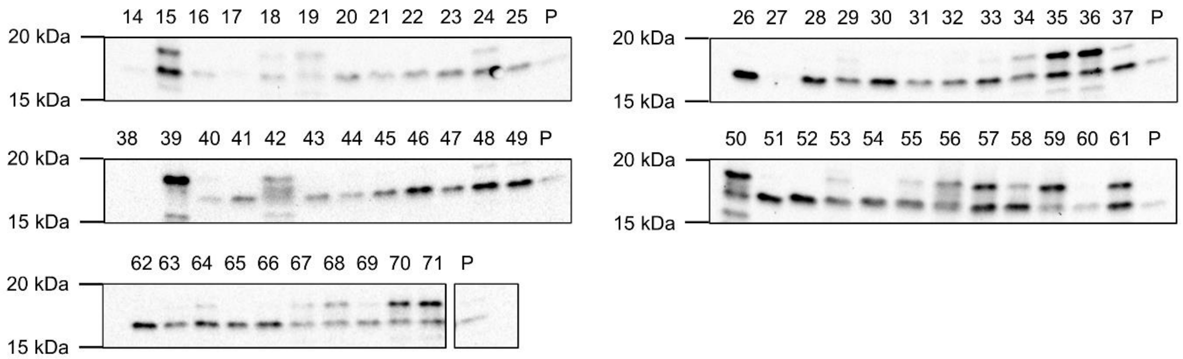

Supplementary Figure 3: Cx26 protein expression in human liver samples. Total protein was extracted from the human liver biopsies (N = 70; n = 1) and used for immunoblotting analysis of Cx26. Immunoblots were visualized with a Pierce™ ECL Western Blotting Substrate kit (Thermo Fisher Scientific, USA) on a ChemiDoc™ MP imaging system (Bio-Rad, USA). Sample numbers are indicated above the blot. (P, pooled control sample)

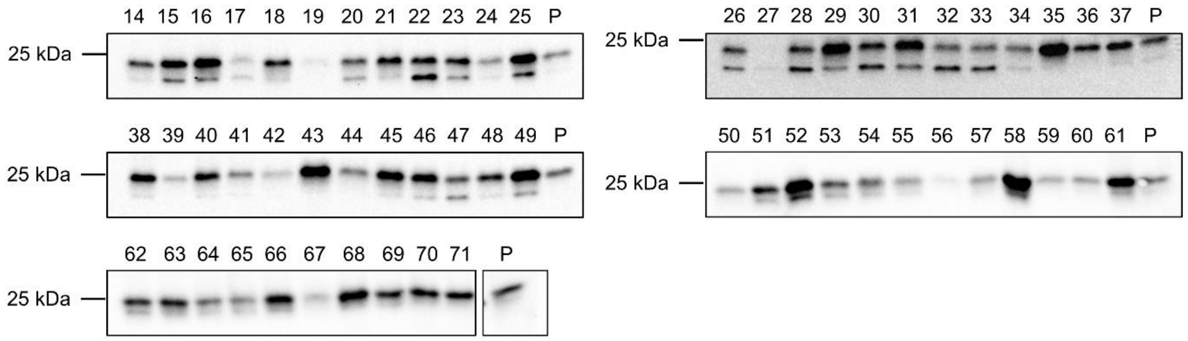

Supplementary Figure 4: Cx32 protein expression in human liver samples. Total protein was extracted from the human liver biopsies (N = 70; n = 1) and used for immunoblotting analysis of Cx32. Immunoblots were visualized with a Pierce™ ECL Western Blotting Substrate kit (Thermo Fisher Scientific, USA) on a ChemiDoc™ MP imaging system (Bio-Rad, USA). Sample numbers are indicated above the blot. (P, pooled control sample)

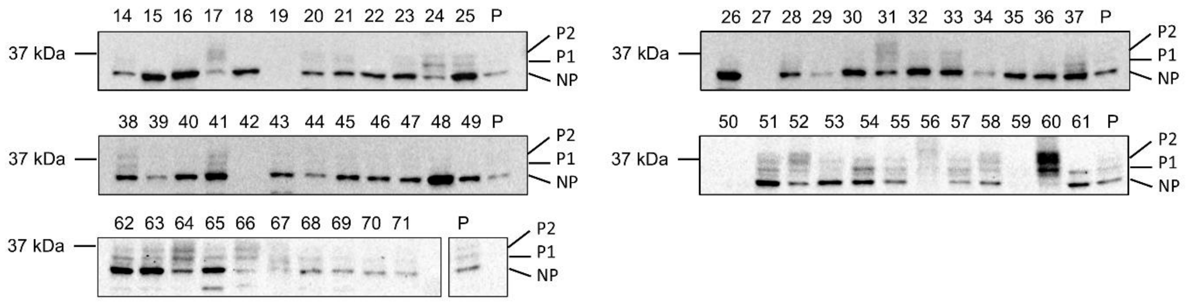

Supplementary Figure 5: Cx43 protein expression in human liver samples. Total protein was extracted from the human liver biopsies (N = 70; n = 1) and used for immunoblotting analysis of Cx43. Immunoblots were visualized with a Pierce™ ECL Western Blotting Substrate kit (Thermo Fisher Scientific, USA) on a ChemiDoc™ MP imaging system (Bio-Rad, USA). Sample numbers are indicated above the blot. (P, pooled control sample; P1 and P2, phosphorylated isoforms; NP, non-phosphorylated isoform)

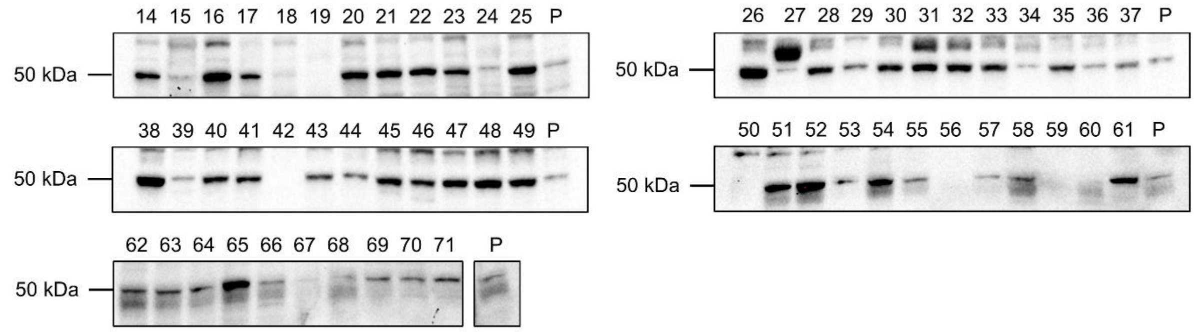

Supplementary Figure 6: Panx1 protein expression in human liver samples. Total protein was extracted from the human liver biopsies (N = 70; n = 1) and used for immunoblotting analysis of Panx1. Immunoblots were visualized with a Pierce^{TM} ECL Western Blotting Substrate kit (Thermo Fisher Scientific, USA) on a ChemiDoc^{TM} MP imaging system (Bio-Rad, USA). Sample numbers are indicated above the blot. (P, pooled control sample)

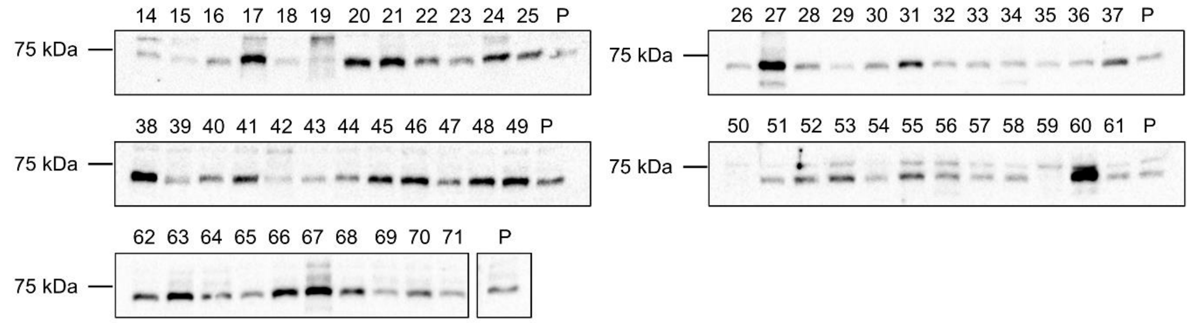

Supplementary Figure 7: Panx2 protein expression in human liver samples. Total protein was extracted from the human liver biopsies (N = 70; n = 1) and used for immunoblotting analysis of Panx2. Immunoblots were visualized with a Pierce^{TM} ECL Western Blotting Substrate kit (Thermo Fisher Scientific, USA) on a ChemiDoc^{TM} MP imaging system (Bio-Rad, USA). Sample numbers are indicated above the blot. (P, pooled control sample)

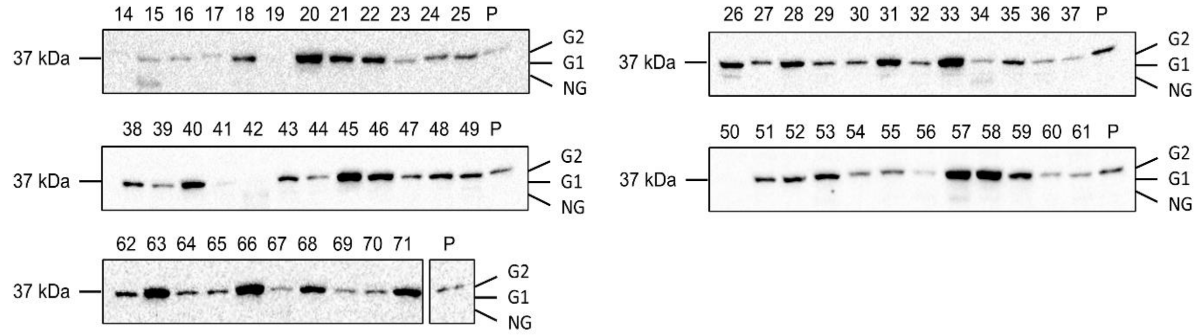

Supplementary Figure 8: Panx3 protein expression in human liver samples. Total protein was extracted from the human liver biopsies (N = 70; n = 1) and used for immunoblotting analysis of Panx3. Immunoblots were visualized with a Pierce^{TM} ECL Western Blotting Substrate kit (Thermo Fisher Scientific, USA) on a ChemiDoc^{TM} MP imaging system (Bio-Rad, USA). Sample numbers are indicated above the blot. (P, pooled control sample; G1 and G2, glycosylated isoforms; NG, non-glycosylated isoform)

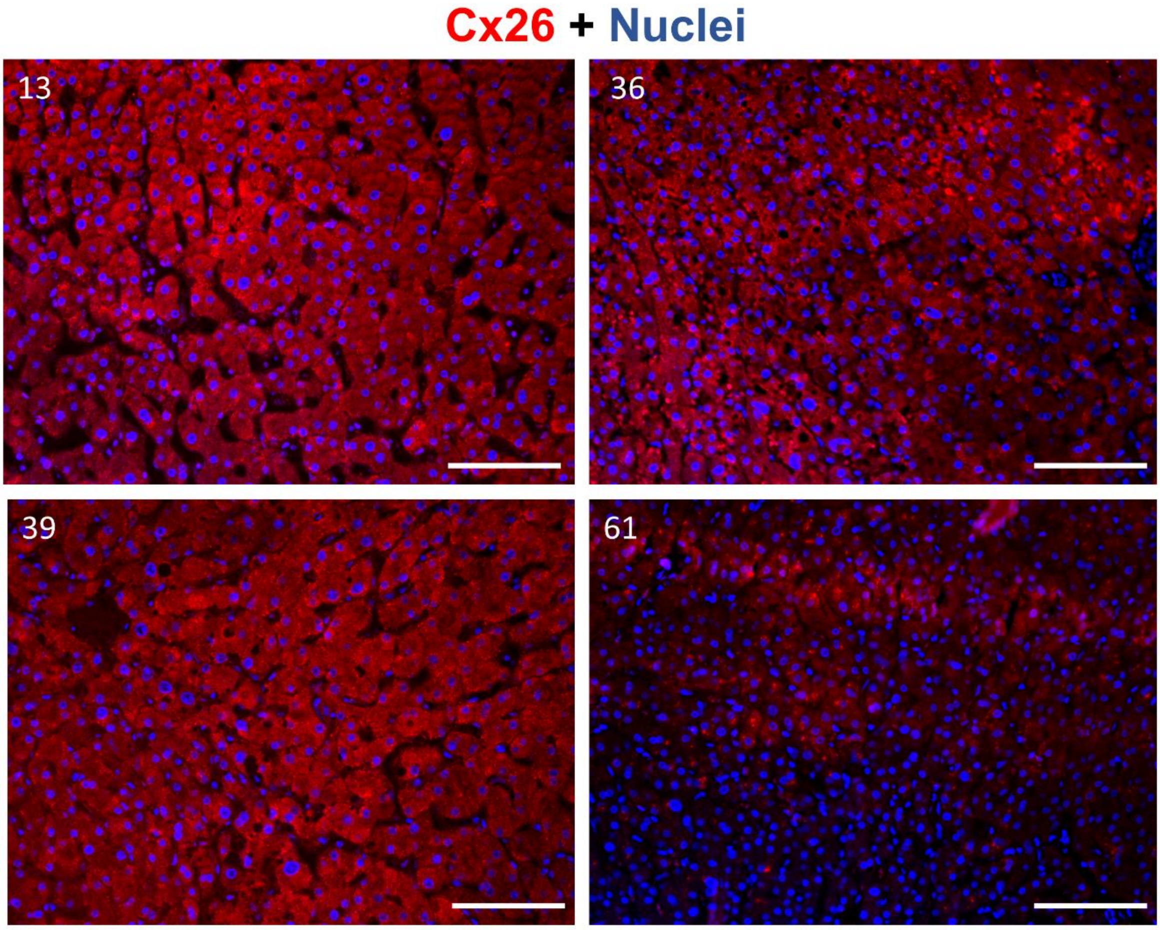

Supplementary Figure 9: Cx26 protein localization in human liver samples. Based on the immunoblot results, 4 samples (S13, S36, S39 and S61) were selected to undergo immunohistochemistry analysis. Paraffin-embedded samples were sectioned into 5 µm thick sections. Cx26 is visualized in red, while the nuclei are counterstained with DAPI. Detection was performed at 20x magnification. Scale bar = 100 µm. Sample numbers are indicated in the left upper corner of the images.

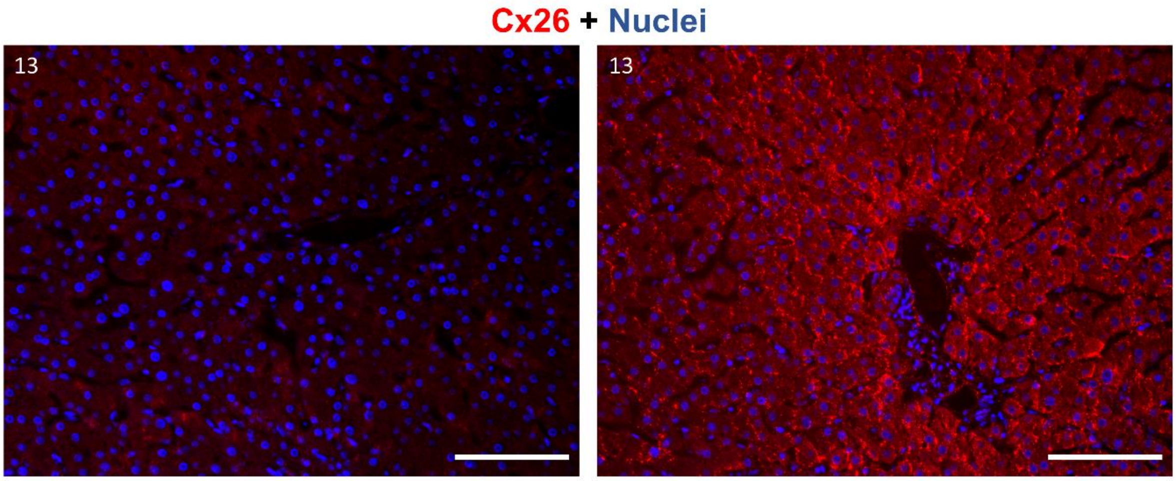

Supplementary Figure 10: Cx26 protein zonation. Based on the immunoblot results, S13 was among the selected samples to undergo immunohistochemistry analysis. It was sectioned into 5 µm thick sections. Cx26 is visualized in red, while the nuclei are counterstained with DAPI. Detection was performed at 20x magnification. Scale bar = 100 µm. Sample numbers are indicated in the left upper corner of the images.

EXCLI Journal 2022;21:1111-1129 – ISSN 1611-2156
Received: June 23, 2022, accepted: August 17, 2022, published: August 22, 2022

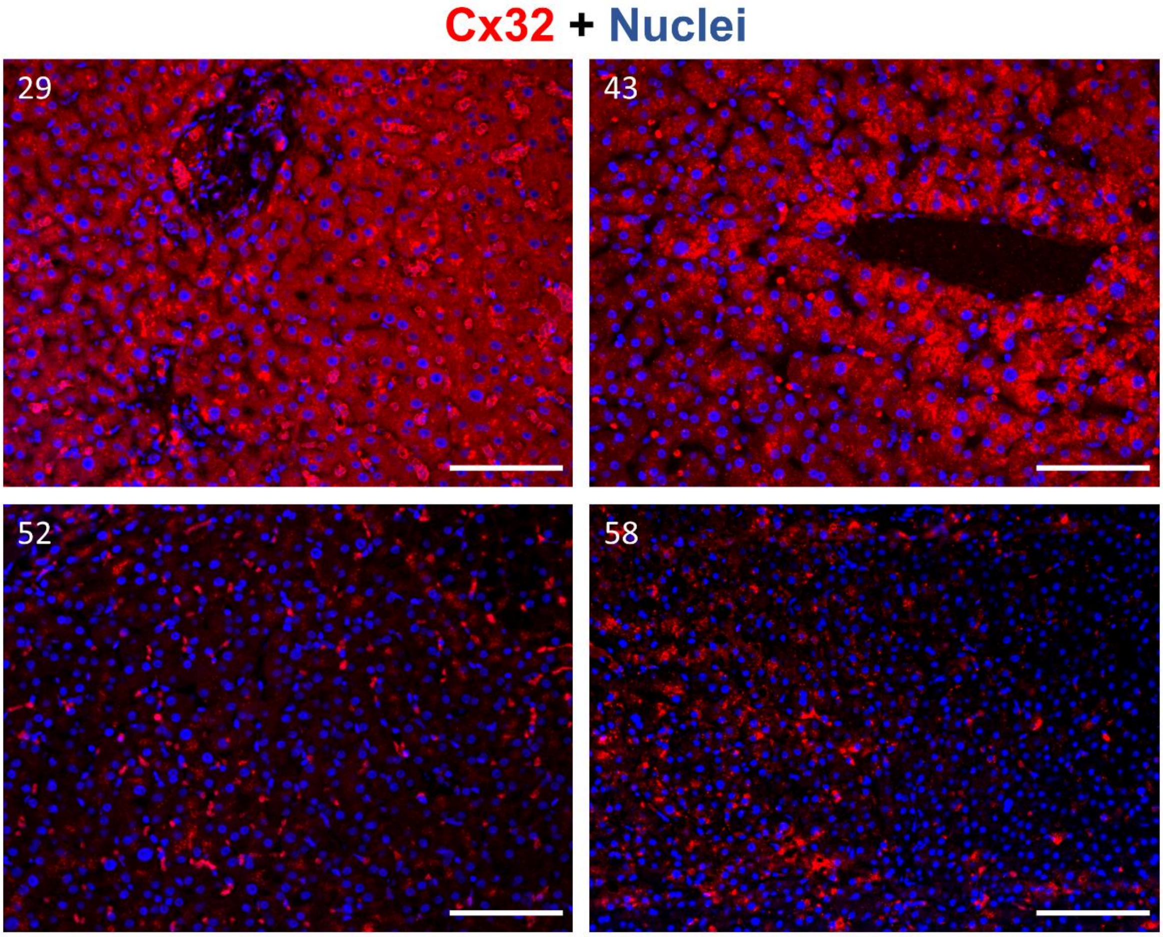

Supplementary Figure 11: Cx32 protein localization in human liver samples. Based on the immunoblot results 4 samples (S29, S43, S52 and S58) were selected to undergo immunohistochemistry analysis. Paraffin-embedded samples were sectioned into 5 µm thick sections. Cx32 is visualized in red, while the nuclei are counterstained with DAPI. Detection was performed at 20x magnification. Scale bar = 100 µm. Sample numbers are indicated in the left upper corner of the images.

EXCLI Journal 2022;21:1111-1129 – ISSN 1611-2156
Received: June 23, 2022, accepted: August 17, 2022, published: August 22, 2022

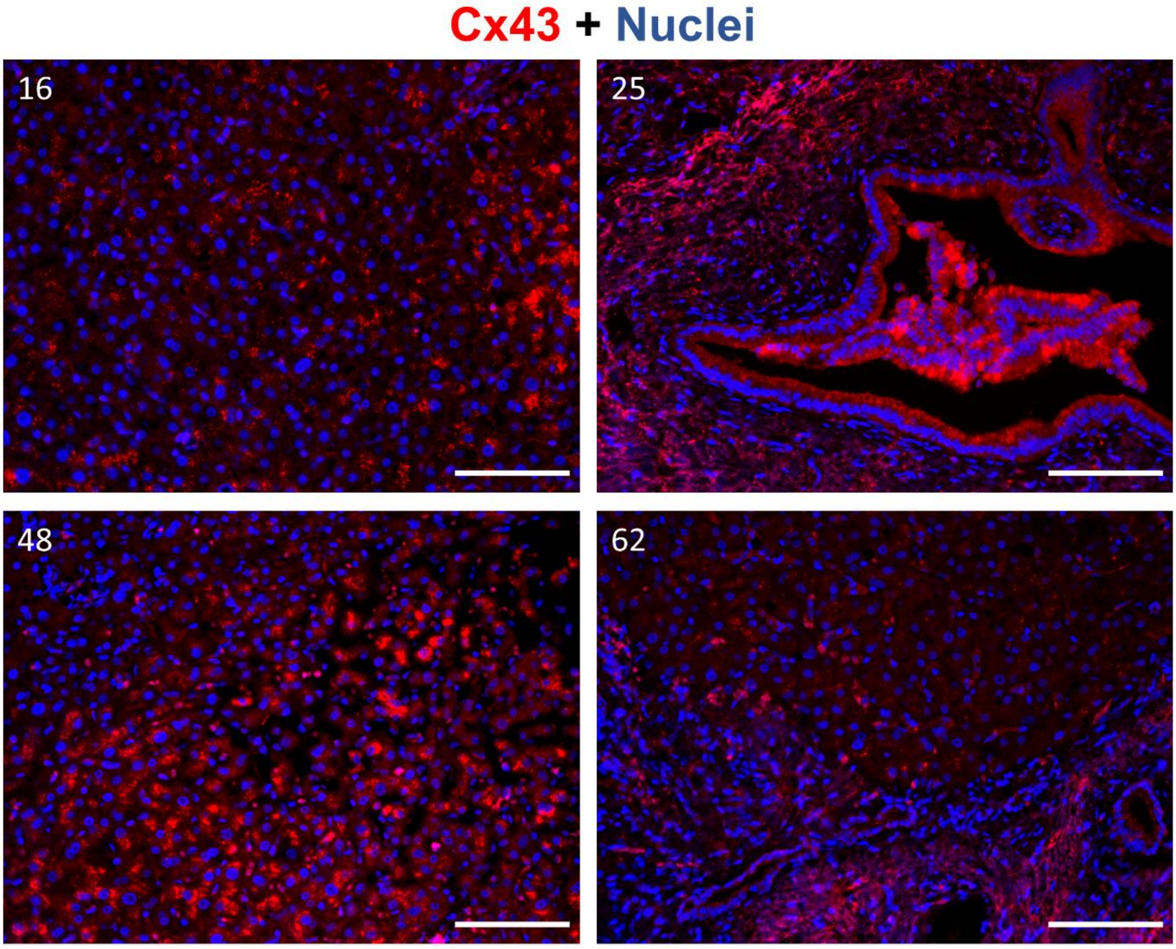

Supplementary Figure 12: Cx43 protein localization in human liver samples. Based on the immunoblot results 4 samples (S16, S25, S48 and S62) were selected to undergo immunohistochemistry analysis. Paraffin-embedded samples were sectioned into 5 µm thick sections. Cx43 is visualized in red, while the nuclei are counterstained with DAPI. Detection was performed at 20x magnification. Scale bar = 100 µm. Sample numbers are indicated in the left upper corner of the images.

EXCLI Journal 2022;21:1111-1129 – ISSN 1611-2156
Received: June 23, 2022, accepted: August 17, 2022, published: August 22, 2022

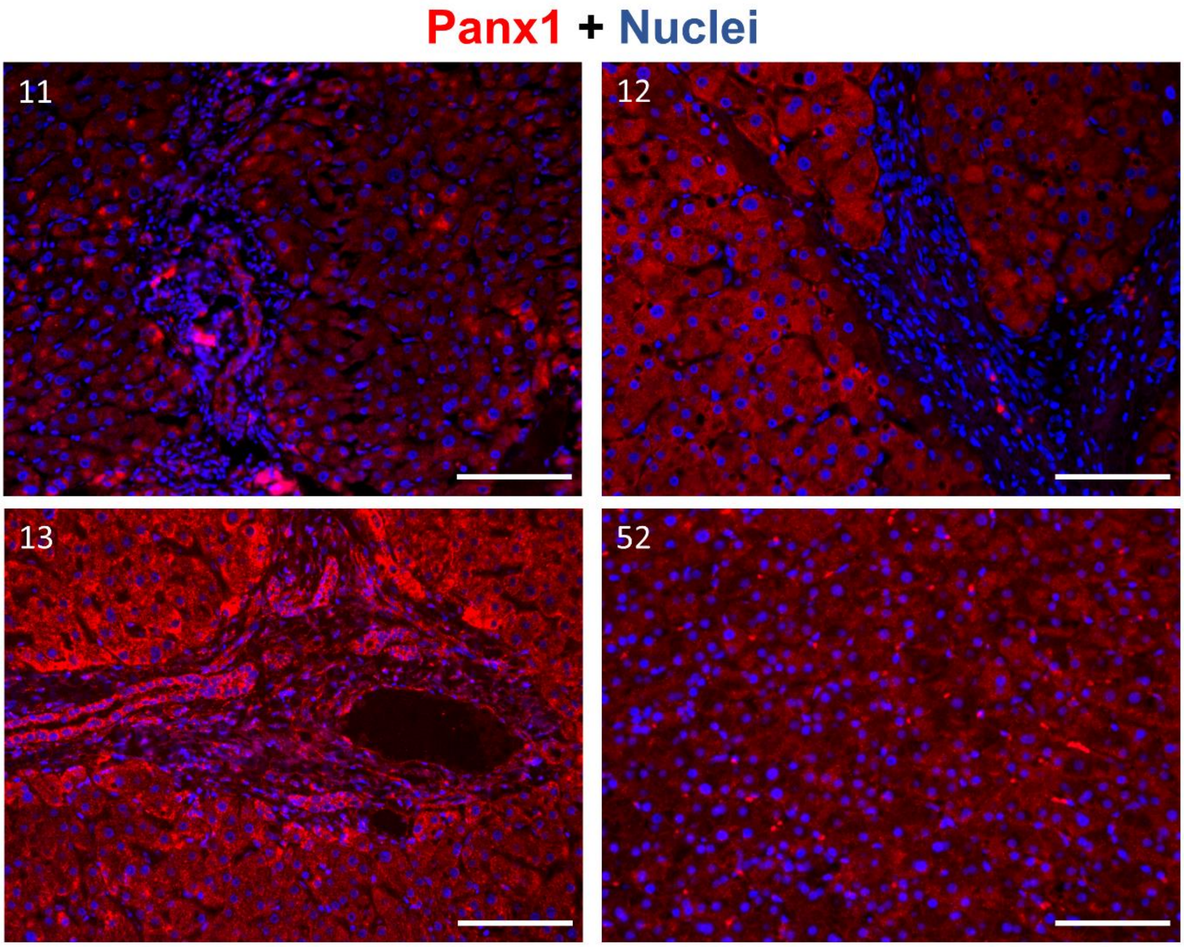

Supplementary Figure 13: Panx1 protein localization in human liver samples. Based on the immunoblot results 4 samples (S11, S12, S13 and S52) were selected to undergo immunohistochemistry analysis. Paraffin-embedded samples were sectioned into 5 µm thick sections. Panx1 is visualized in red, while the nuclei are counterstained with DAPI. Detection was performed at 20x magnification. Scale bar = 100 µm. Sample numbers are indicated in the left upper corner of the images.

EXCLI Journal 2022;21:1111-1129 – ISSN 1611-2156
Received: June 23, 2022, accepted: August 17, 2022, published: August 22, 2022

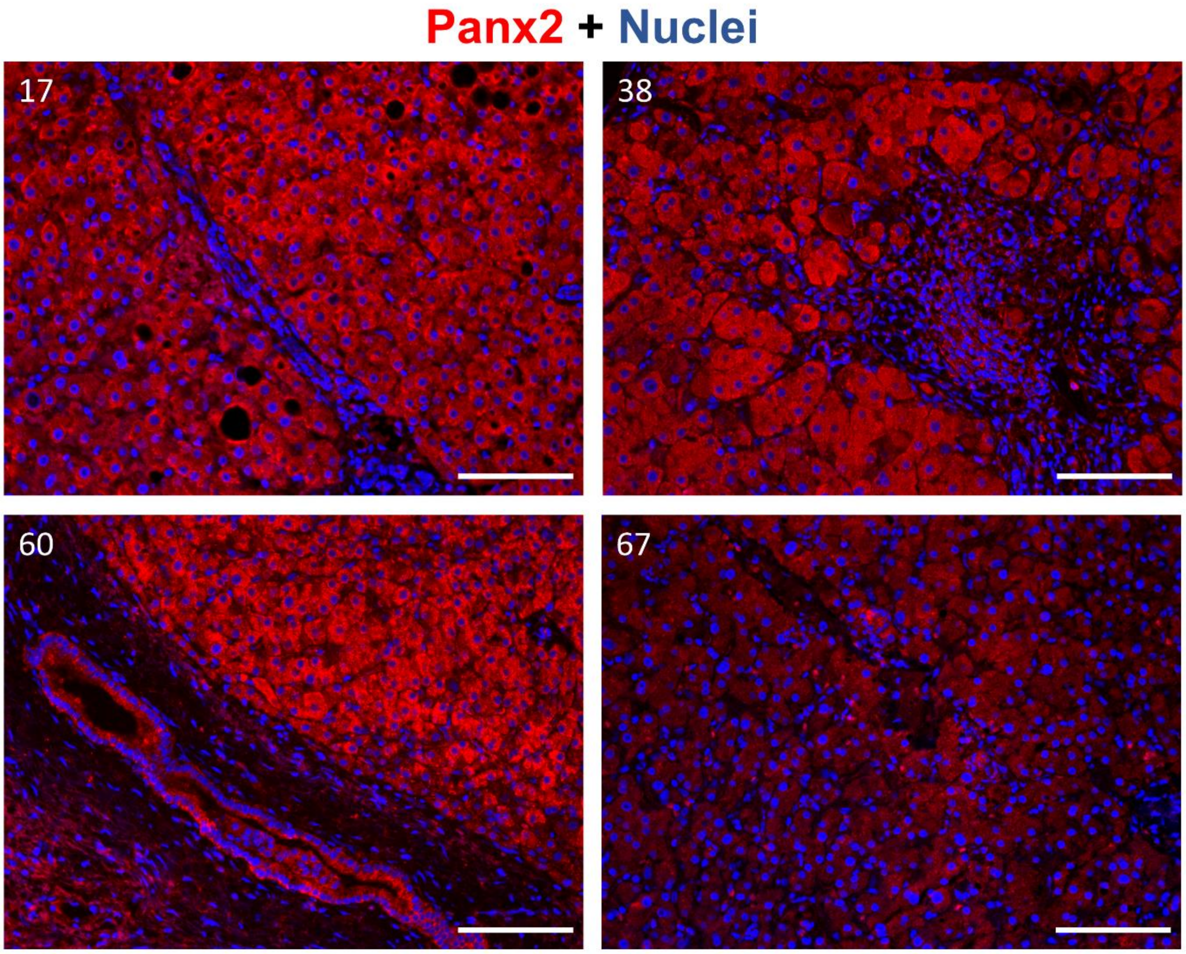

Supplementary Figure 14: Panx2 protein localization in human liver samples. Based on the immunoblot results 4 samples (S17, S38, S60 and S67) were selected to undergo immunohistochemistry analysis. Paraffin-embedded samples were sectioned into 5 µm thick sections. Panx2 is visualized in red, while the nuclei are counterstained with DAPI. Detection was performed at 20x magnification. Scale bar = 10 µm. Sample numbers are indicated in the left upper corner of the images.

EXCLI Journal 2022;21:1111-1129 – ISSN 1611-2156
Received: June 23, 2022, accepted: August 17, 2022, published: August 22, 2022

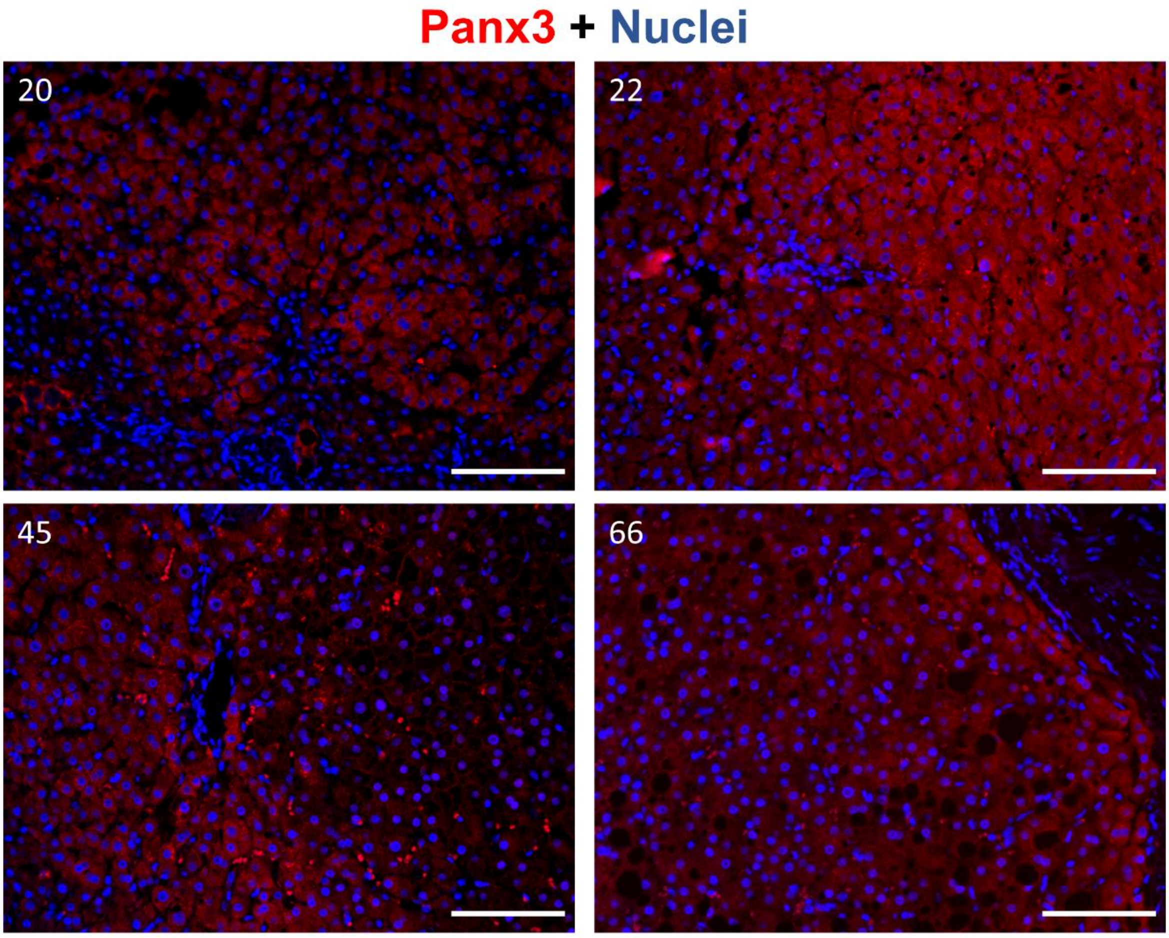

Supplementary Figure 15: Panx3 protein localization in human liver samples. Based on the immunoblot results 4 samples (S20, S22, S45 and S66) were selected to undergo immunohistochemistry analysis. Paraffin-embedded samples were sectioned into 5 µm thick sections. Panx3 is visualized in red, while the nuclei are counterstained with DAPI. Detection was performed at 20x magnification. Scale bar = 100 µm. Sample numbers are indicated in the left upper corner of the images.
